# Serum Uric Acid Is Independently Associated with Risk of Obstructive Sleep Apnea-Hypopnea Syndrome in Chinese Patients with Type 2 Diabetes

**DOI:** 10.1155/2019/4578327

**Published:** 2019-04-03

**Authors:** Caiyu Zheng, Haiqu Song, Shunhua Wang, Jing Liu, Tingting Lin, Chunmin Du, Huan Xie, Zhongyun Chen, Silan Zheng, Zhibin Li, Xuejun Li, Changqin Liu

**Affiliations:** ^1^School of Medicine, Xiamen University, Xiamen, China; ^2^Department of Endocrinology and Diabetes, The First Affiliated Hospital of Xiamen University, Xiamen, China; ^3^The Fujian University of Traditional Chinese Medicine, Fuzhou, China; ^4^Xiamen Diabetes Institute, The First Affiliated Hospital, Xiamen University, Xiamen, China; ^5^Epidemiology Research Unit, The First Affiliated Hospital, Xiamen University, Xiamen, China; ^6^Department of Endocrinology and Diabetes, The Teaching Hospital of Fujian Medical University, Xiamen, China

## Abstract

**Purpose:**

We aimed to investigate the association between serum uric acid (SUA) levels and obstructive sleep apnea-hypopnea syndrome (OSAHS) in patients with type 2 diabetes.

**Methods:**

A cross-sectional study of 212 type 2 diabetes mellitus (T2DM) patients was conducted in Xiamen, China. All patients underwent polysomnography (PSG) recordings for OSAHS diagnosis. Patients were grouped according to the apnea-hypopnea index (AHI) as mild (5-14.9), moderate (15-29.9), and severe (≧30) OSAHS. Patients with AHI ≤ 4.9 served as the control group. Weight, body mass index (BMI), SUA, liver function, renal function, blood pressure, lipid profiles, and glycemic parameters were measured.

**Results:**

A total of 158 patients (101 men and 57 women) with complete data were analyzed in this study. 127 patients were identified as OSAHS. Among the 127 patients with OSAHS, 56 (44.1%), 37 (29.1%), and 34 (26.8%) had mild, moderate, and severe OSAHS, respectively. Correlation analyses showed that the SUA level was significantly related to the apnea-hypopnea index (AHI) (*r* = 0.194, *p* = 0.016). The level of SUA was significantly higher among OSAHS patients compared to the control group (control group: 333.14 ± 80.52 *μ*mol/L, mild group: 345.50 ± 90.27 *μ*mol/L, moderate group: 363.59 ± 134.26 *μ*mol/L, and severe group: 428.37 ± 123.58 *μ*mol/L and *p* = 0.029). Multivariable logistic regression analyses showed that SUA was the independent risk factor for OSAHS (OR: 1.006, 95% CI: 1.001-1.011, *p* = 0.020).

**Conclusions:**

The SUA level is significantly associated with the severity of OSAHS and should be controlled when managing OSAHS.

## 1. Introduction

In recent decades, serum uric acid (SUA), which is the end product of purine metabolism in humans, has been consistently found to predict the development of metabolic syndrome (MetS) [1, 2]. Growing epidemiological studies suggested that hyperuricemia might be one component of MetS [3]. Previous studies suggested that the SUA level may be a useful predictor for metabolic disorders [4]. With the increase of SUA levels, the prevalence of obesity significantly increased. The SUA level is independently related to obesity in type 2 diabetes mellitus (T2DM) even after adjusting for other obesity risk factors [5, 6].

Obstructive sleep apnea-hypopnea syndrome (OSAHS) is a syndrome characterized by recurrent episodes of shallow or paused breathing during sleep and normally leads to intermittent hypoxia (IH) and sleep disruption due to partial or complete obstructions of the upper airway during sleep. OSAHS affects 3-7% of men and 2-5% women in the general population [7]. OSAHS becomes a major public health burden and is associated with important medical consequences. A large body of evidence has identified OSAHS as an independent risk factor for cardiovascular morbidity [8]. Increasing evidence also demonstrates the close association between obesity and OSAHS [9]. Obesity is a major pathogenetic factor for OSAHS [10].

Previous studies observed an association between SUA levels and OSAHS. In a population-based survey study, a strong association was found between SUA and OSAHS even after adjustment for confounding factors such as gender, age, and BMI [11]. In a large set of OSAHS patients without known comorbidities, SUA is also independently associated with OSAHS severity [12]. Kanbay et al. found that hyperuricemia (HUA) is significantly associated with cardiovascular disease in patients with OSAHS even after adjusting for traditional risk factors for cardiovascular disease [13]. Kosacka et al. found that OSAHS patients with increased SUA concentration have a higher risk of atherosclerosis and a higher prevalence of cardiovascular events [14]. However, among the individuals with T2DM, the relationship between the SUA levels and the severity of OSAHS remained unclear. So, the current study is aimed at exploring whether the SUA level was significantly associated with OSAHS in T2DM patients.

## 2. Methods

### 2.1. Ethics Statement

The study was approved by the Human Research Ethics Committee of the Xiamen First Hospital. All subjects provided written informed consent.

### 2.2. Participants

**Figure 1:**
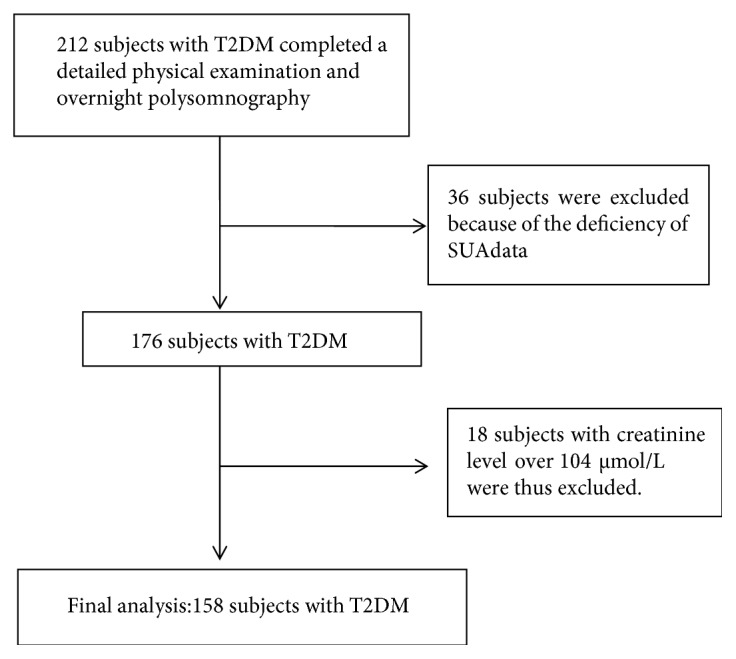
Flow diagram.

A total of 212 adult subjects with T2DM were recruited from the First Affiliated Hospital, Xiamen University, China, from June 2015 to June 2017. These subjects underwent face-to-face interview with uniform questionnaires. Subjects were admitted for routine check-up evaluations and underwent a detailed physical examination and overnight polysomnography (PSG). Exclusion criteria included the presence of hyperthyroidism or hypothyroidism, acute illnesses, serious heart diseases, uncontrolled hypertension, presence of cancer, craniofacial abnormalities, any respiratory disorder other than OSAHS, and current use of hypnotics or any treatment for breathing disorders. Among the eligible patients, 36 subjects were excluded because of the deficiency of SUA data. 18 patients with the creatinine level over 104 *μ*mol/L were further excluded from the study. The remaining 158 patients (101 men and 57 women) were left for analyses. The flow diagram is shown in Figure 1.

### 2.3. Anthropometric and Biochemical Measurements

Anthropometric measurements include body weight, height, waist circumference (WC), blood pressure (BP), body mass index (BMI), and neck circumference (NC). NC was measured at the middle of the neck between the midcervical spine and the midanterior neck 0.5 cm below the laryngeal prominence. BMI was calculated as the weight in kilograms divided by the square of the height in meters. WC was measured at the midpoint between the inferior costal margin and the superior border of the iliac crest on the midaxillary line. Weight and height were measured with light clothes and without shoes to the nearest 0.1 kg and 0.5 cm, respectively.

All blood samples were obtained after 12-hour fasting. Blood samples were tested in the central laboratory of the First Affiliated Hospital, Xiamen University, as described in details previously [15]. Briefly, triglyceride (TG), total cholesterol (TC), high-density lipoprotein cholesterol (HDL-c), low-density lipoprotein cholesterol (LDL-c), aspartate aminotransferase (AST), and alanine aminotransferase (ALT) were determined on a HITACHI 7450 analyzer (HITACHI, Tokyo, Japan). Serum creatinine (Scr) and SUA were measured with an autoanalyzer (COBAS INTEGRA 400 plus, Roche, Basel, Switzerland). Serum fasting C peptide was measured by electrochemiluminescence immunoassay (Elecsys, Roche), and hemoglobin A1c (HbA1c) were measured by the Bio-Rad VARIANT Hemoglobin A1c assay. HUA was defined as the serum uric acid level > 7.0 mg/dL in males and >6.0 mg/dL in females [15].

### 2.4. Polysomnography

Polysomnography (PSG) is the gold standard method for diagnosing and assessing the severity of OSAHS. All patients underwent an overnight PSG study performed from 11:00 pm to 7:00 am according to standard techniques with monitoring of the electroencephalogram (EEG), electrooculogram (EOG), electromyogram (EMG), flow, thoracic and abdominal respiratory effort, oximetry, and body position. All PSG data were collected and stored using an E-Series digital system (Compumedics Ltd., Australia). Polysomnographic recordings were interpreted in accordance with the current American Academy of Sleep Medicine (AASM) guidelines [16]. The recording duration ≥ 5 h was required for validation, and monitoring was repeated on a second night if subjective sleep latency exceeded 2 h on the first night or if respiratory parameters were missing. Polysomnographic records were scored according to standard criteria. The apnea-hypopnea index (AHI) was defined as the total number of obstructive apnea and hypopnea per hour of sleep, and the severity of OSAHS was determined by AHI. Absence of OSAHS or mild, moderate, and severe degrees were defined by an AHI of ≤4.9, AHI of ≤5-14.9, AHI of ≤15-29.9, and AHI of ≧30 events/hour, respectively [17].

### 2.5. Statistical Analysis

Data were analyzed with the use of IBM SPSS Statistics 21.0. Results are expressed as mean ± standard deviation (SD). Differences between groups were analyzed using ANOVA or the Mann-Whitney *U* test for continuous variables and the chi-square test for categorical variables. The correlation of SUA with AHI was analyzed using Pearson's correlation analysis. Multivariable logistic regression analysis was used to calculate the odds ratio (OR) for OSAHS in different models with adjustment for potential confounders. In model 1, no variables were adjusted for. In model 2, age, sex, BMI, and the waist/hip were adjusted for. In model 3, hypertension and dyslipidemia plus model 2 were adjusted for. All *p* values presented are two-tailed, and values less than 0.05 are considered statistically significant.

## 3. Results

**Figure 2:**
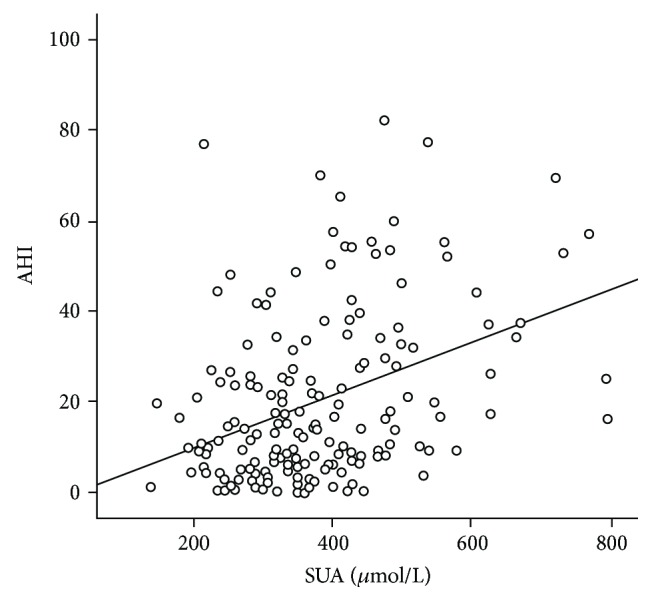
Correlations of SUA levels with AHI in the subjects.

The baseline characteristics of the studied subjects are shown in Table 1. The mean age of the participants was 51.55 ± 13.43 years. The average BMI, WC, and HbA1c were 28.30 ± 4.85 kg/m^2^, 0.97 ± 0.09 m, and 9.88 ± 2.45%, respectively. The overall prevalence rate of OSAHS was 80.37% in type 2 diabetes mellitus patients in the current cohort. Among the 127 patients with OSAHS, 56 (44.1%), 37 (29.1%), and 34 patients (26.8%) presented mild, moderate, and severe OSAHS, respectively. Comparing between the four groups (ordering as none, mild, moderate, and severe OSAHS), there were significant increases in BMI (27.29 ± 4.51 kg/m^2^, 26.21 ± 3.50 kg/m^2^, 28.97 ± 5.56 kg/m^2^, and 31.76 ± 4.25 kg/m^2^, respectively, *p* < 0.05), NC (38.68 ± 3.79 cm, 38.15 ± 3.17 cm, 40.12 ± 4.03 cm, and 42.48 ± 3.22 cm, respectively, *p* < 0.05), and SUA (333.14 ± 80.52 *μ*mol/L, 345.50 ± 90.27 *μ*mol/L, 363.59 ± 134.26 *μ*mol/L, and 428.37 ± 123.58 *μ*mol/L, respectively, *p* = 0.029). Pearson's correlation coefficients with adjustment for gender, age, and BMI are shown in Figure 2 and show that serum SUA levels were positively correlated with AHI (*r* = 0.194, *p* = 0.016).

Table 2 shows the characteristics of the subjects stratified by hyperuricemia. Subjects with hyperuricemia had significantly higher BMI (30.28 ± 5.95 kg/m^2^ vs. 27.36 ± 3.90 kg/m^2^, *p* = 0.002) and TG (2.67 ± 2.16 mmol/L vs. 1.94 ± 1.43 mmol/L, *p* = 0.027) levels compared with the control groups.

Table 3 shows the adjusted ORs with associated 95% confidence interval (CI) of OSAHS by using multivariable logistic regression. Model 1 is a univariable model. In model 2, the confounding factors of age, sex, BMI, and the waist/hip ratio were adjusted for. In model 3, the confounding factors of age, sex, BMI, the waist/hip ratio, hypertension, and dyslipidemia were adjusted for. In all 3 models, SUA was significantly associated with OSAHS. In model 3, the adjusted OR with associated 95% CI of SUA with OSAHS was 1.006 (1.001–1.011; *p* < 0.01).

## 4. Discussion

The study showed a significant association between SUA and OSAHS in the patients with T2DM. The results suggested that type 2 diabetic patients with OSAHS had significantly higher levels of SUA than those without OSAHS (428.37 ± 123.58 *μ*mol/L vs. 333.14 ± 80.52 *μ*mol/L, *p* = 0.029). Furthermore, the SUA level was significantly correlated to the severity of OSAHS, with the Pearson's correlation coefficients between SUA levels and AHI of 0.194 (*p* = 0.016). With adjustment for potential confounding factors such as gender, age, and BMI, logistic regression analysis showed that SUA was independently associated with risk of OSAHS in patients with T2DM.

Similar to our data, a previous study found that after adjustment for confounding factors such as gender, age, BMI, social class, ethnicity, cholesterol, triglycerides, blood pressure, and glucose, the patients with OSAHS had higher levels of SUA than those without OSAHS [11]. It showed that OSAHS was also independently associated with increased risk of SUA. An increase in 1 *μ*mol/L in the SUA level was associated with 16% increased risk of OSAHS (95% CI = 1.01-1.33). The OR is higher than we found in the current study, likely due to the different populations studied. In our study, we had excluded the patients with the serum creatinine level over 104 *μ*mol/L and all the subjects were type 2 diabetes mellitus patients.

Over the past few decades, hyperuricemia has been found to be a risk factor for atherosclerosis and hypertension [18]. One of the mechanisms is the oxidative stress [19]. The epidemic of hyperuricemia, obesity, and cardiovascular and cerebrovascular diseases has cast a heavy burden on the world [20, 21]. Also, a large body of evidence has identified that increasing SUA levels could be associated with MetS [22, 23]. In a previous study, Liu et al. [15] found that an increased SUA level was significantly related with increased prevalence rates of insulin resistance and MetS. In a recent study, Li et al. found that BMI significantly increased with the increase of SUA levels in T2DM [24].

In the clinic, obesity is associated with insulin resistance and increases the risk for type 2 diabetes [25]. But there is no consensus for a unifying mechanism of insulin resistance. Of all possible mechanisms, inflammation has been the major concept. Obesity contributes to the initiation of chronic inflammation, and inflammation inhibits the insulin signaling activity in hepatocytes and adipocytes [26]. Another cross-sectional study with larger samples shows that the systemic inflammation including high-sensitivity C-reactive protein (hs-CRP), fibrinogen, erythrocyte sedimentation rate (ESR), and SUA significantly increases in OSAHS patients without known comorbidities and correlates with OSA severity [12].

It was well known that obese individuals present higher incidence of OSAHS, which contributes to increased morbidity and mortality [27, 28]. There is a strong relationship between obesity and OSAHS [29]. A previous study suggests that, in obese individuals, OSAHS is independently associated with inflammation and insulin resistance [30]. So our data indicate that the increased SUA level was an independent risk factor for the severity of OSAHS, possibly by insulin resistance. But it needs more studies to validate the hypothesis.

Our study also had a few limitations. First, the causal relationship between SUA and OSAHS could not be determined because of the cross-sectional study design. Second, the number of patients in this study was relatively small; therefore, our results should be confirmed in future prospective cohort studies with larger sample sizes. Third, the present subjects were not randomly sampled and the selection bias was obvious, so our results should be interpreted with much caution.

## 5. Conclusion

The SUA level is significantly associated with the severity of OSAHS and should be considered in OSAHS management in the clinic.

## Figures and Tables

**Table 1:** Characteristics of the control group and mild, moderate, and severe OSAHS groups stratified by the apnea-hypopnea index (AHI).

	AHI ≤ 4.9	AHI ≤ 5~14.9	AHI ≤ 15~29.9	AHI ≥ 30	*p* value
No.	31	56	37	34	—
Gender (male/female)	19/12	34/22	21/16	27/7	0.251
Age (years)	44.87 ± 13.97	54.59 ± 11.63	54.00 ± 14.49	50.03 ± 12.63	0.018
BMI (kg/m^2^)	27.29 ± 4.51	26.21 ± 3.50	28.97 ± 5.56	31.76 ± 4.25	≤0.001
Waist/hip	0.94 ± 0.06	0.96 ± 0.11	0.98 ± 0.07	0.99 ± 0.06	0.391
NC (cm)	38.68 ± 3.79	38.15 ± 3.17	40.12 ± 4.03	42.48 ± 3.22	0.003
T2DM (years)	3.47 ± 5.42	8.07 ± 6.99	5.18 ± 6.22	4.82 ± 5.90	0.099
Systolic BP (mmHg)	124.06 ± 17.27	130.02 ± 15.63	130.41 ± 13.75	134.20 ± 20.09	0.452
Diastolic BP (mmHg)	77.39 ± 8.55	76.14 ± 11.06	79.16 ± 9.29	80.11 ± 11.31	0.804
SUA (*μ*mol/L)	333.14 ± 80.52	345.50 ± 90.27	363.59 ± 134.26	428.37 ± 123.58	0.029
C peptide (ng/mL)	1.15 ± 0.68	1.22 ± 0.81	1.73 ± 1.11	2.37 ± 2.30	0.024
HbA1c (%)	10.81 ± 2.37	10.02 ± 2.50	9.96 ± 2.40	8.79 ± 2.19	0.049
HDL-c (mmol/L)	1.07 ± 0.23	1.08 ± 0.19	1.06 ± 0.23	1.01 ± 0.22	0.793
LDL-c (mmol/L)	3.36 ± 0.89	2.96 ± 0.99	3.11 ± 1.11	3.07 ± 1.01	0.480
Triglyceride (mmol/L)	1.77 ± 1.13	2.36 ± 2.30	2.31 ± 1.53	2.09 ± 1.23	0.935
TC (mmol/L)	5.26 ± 1.06	5.59 ± 3.4	5.30 ± 1.11	5.03 ± 1.07	0.552
AST (U/L)	23.60 ± 17.96	19.54 ± 13.01	23.64 ± 15.22	22.49 ± 8.54	0.630

Data was expressed as mean ± SD; BMI: body mass index; NC: neck circumference; SUA: serum uric acid; HDL-c: high-density lipoprotein cholesterol; LDL-c: low-density lipoprotein cholesterol; HbA1c: hemoglobin A1c; TC: total cholesterol; AST: aspartate aminotransferase.

**Table 2:** Characteristics of the subjects stratified by hyperuricemia.

	HUA	NUA	*p* value
Gender (male/female)	70/22	31/35	0.324
Age (years)	47.66 ± 15.28	53.66 ± 11.95	0.008
BMI (kg/m^2^)	30.28 ± 5.95	27.36 ± 3.90	0.002
Waist/hip	0.96 ± 0.07	0.97 ± 0.09	0.386
Neck circumference (cm)	39.99 ± 4.13	39.45 ± 3.72	0.496
Duration of T2DM (years)	5.12 ± 6.41	6.17 ± 6.48	0.334
Systolic BP (mmHg)	130.75 ± 15.43	128.88 ± 16.65	0.494
Diastolic BP (mmHg)	80.40 ± 10.13	76.35 ± 9.44	0.014
C peptide (ng/mL)	1.82 ± 1.15	1.45 ± 1.44	0.136
HbA1c (%)	9.44 ± 2.30	10.15 ± 2.47	0.087
HDL-c (mmol/L)	1.02 ± 0.189	1.08 ± 0.218	0.081
LDL-c (mmol/L)	2.95 ± 1.08	3.16 ± 0.97	0.223
Triglyceride (mmol/L)	2.67 ± 2.16	1.94 ± 1.43	0.028
Total cholesterol (mmol/L)	5.25 ± 1.18	5.38 ± 2.57	0.709
AST (U/L)	24.46 ± 14.41	20.73 ± 13.51	0.113

Data was expressed as mean ± SD; BMI: body mass index; HDL-c: high-density lipoprotein cholesterol; LDL-c: low-density lipoprotein cholesterol; HbA1c: hemoglobin A1c; AST: aspartate aminotransferase; HUA: hyperuricemia; NUA: normal serum uric acid.

**Table 3:** To investigate the role of SUA in predicting OSAHS, logistic regression was performed.

	OR	CI (95%)	*p* value
Model 1	1.005	1.001-1.009	0.014
Model 2	1.006	1.001-1.011	0.013
Model 3	1.006	1.001-1.011	0.020

Model 1: crude model; model 2: adjusted for age, sex, BMI, and waist/hip; model 3: adjusted for age, sex, BMI, waist/hip, hypertension, and dyslipidemia.

## Data Availability

The data used to support the findings of this study are available from the corresponding author upon reasonable request.
